# Supplementary material for: Direct Simulations of H–He Mixtures at Planetary Interior Conditions: Demixing, Insulator–Metal Transition and Miscibility Boundaries
Source: J Phys Chem Lett. 2026 Jun 4;17(24):6620–6. doi: 10.1021/acs.jpclett.6c00924 (PMC13288631; doi:10.1021/acs.jpclett.6c00924)
Supplement: Supplementary file 1 [file jz6c00924_si_001.pdf]

Supporting Information - "Direct simulations of H–He mixtures at planetary interior conditions: demixing, insulator–metal transition and miscibility boundaries"

Valentin V. Karasiev,[1,*] S. X. Hu,[1,2,3] Joshua P. Hinz,[4] R. M. N. Goshadze,[1] Shuai Zhang,[1] Armin Bergermann,[5] and Ronald Redmer[5]

[1] *Laboratory for Laser Energetics, University of Rochester, 250 East River Road, Rochester, New York 14623 USA*

[2] *Department of Physics and Astronomy, University of Rochester, NY 14627, USA*

[3] *Department of Mechanical Engineering, University of Rochester, NY 14627, USA*

[4] *Laboratory for Laser Energetics, University of Rochester, 250 East River Road, Rochester, New York 14623 USA; Current Address: Physics Department Southern University, 801 Harding Blvd, Baton Rouge, LA 70807, USA*

[5] *Institut für Physik, Universität, D-18051 Rostock, Germany*

COMPUTATIONAL DETAILS

All large-scale AIMD simulations in the isothermal-isobaric NPT ensemble were performed by using the plane-wave finite-temperature DFT Vienna *ab initio* Simulation Package (VASP) [1] with the projector augmented wave (PAW) method [2] modeling the electron-ion electrostatic interaction. Thermal XC effects were considered using the Karasiev-Dufty-Trickey GGA level functional [3]. Simulations were performed for systems with 1024 electrons for two helium fractions, x = 0.11304 ($He_{104}H_{816}$) and x = 0.27522 ($He_{221}H_{582}$). We used a plane wave cutoff of 1400 eV. In all simulations, the Baldereschi mean-value k-point (BMVP) [4] was used to sample the Brillouin zone of (near-)cubic supercells. The number of thermally occupied bands included in our simulations was large enough to ensure the highest energy state is occupied around 0.2×10^{-5} or below. The MD time step, depending on temperature, varied between 0.2 and 1.0 fs. After reaching equilibrium (usually 5000 MD steps), we run our simulations for up to $40,000$ MD steps. We followed Ref. [5] for AIMD simulations of pure H. We conducted simulations along two isobars using the ground-state Perdew-Burke-Ernzerhof XC functional [6] (see Fig. S5) for a He fraction of x = 0.11304 to estimate the thermal XC effects on the demixing boundary.

We performed convergence tests with respect to the system size by reducing the number of particles by a factor of two. We found that the behavior of the magnitude of the first peak in the H-He RDF along the selected isobar remains unchanged providing the same temperature of mixing (see Sec. below). When we start simulations from a perfectly mixed configuration at conditions when the system is expected to be demixed, the system demixes within 2.5-5.0 ps, demixing in many cases can be observed by a visual inspection of simulation cell snapshots. We keep running AIMD for up to 30 ps, and the system remains in the same demixed phase. This clearly demonstrates the convergence with respect to the AIMD time length.

Electrical and thermal conductivities were computed using the Kubo-Greenwood formalism implemented in the VASP package. A typical number of statistically independent snapshots used for calculations along AIMD trajectories was between 100 and 150 for mixtures. Contrary, we used 21 snapshots for pure H. We performed a set of convergence tests with respect to the broadening parameter δ, and k-mesh (BMVP results have been compared to the 2x2x2 $k-$mesh sampling). Comparisons between the Gaussian and Lorentzian broadening [7] were performed at few selected conditions. Eventually, Kubo-Greenwood calculations were performed with the thermal KDT16 XC functional, the Gaussian broadening with $\delta = 0.40$ eV, the BMVP, and the number of bands increased by a factor of three (compared to the number used in AIMD simulations).

JUSTIFICATION FOR THE CHOICE OF THE THERMAL KDT16 XC FUNCTIONAL

It is known that AIMD simulations with the GGA-level ground-state PBE XC and classical treatment of ions predict reasonably well the first-order liquid-liquid phase transition and related structural properties in dense hydrogen (see results in Refs. [5, 8]). Reasonable accuracy is reached due to partial cancellations between missing nuclear quantum effects (NQEs) and proper inaccuracies of the functional. Thermal KDT16 XC, by construction, reduces to the ground-state PBE at low-T. Hence, the accuracy of the thermal KDT16 XC predictions, at low-T, is near-identical to the accuracy of PBE predictions. Thermal XC effects, that KDT16 takes into account, might be not negligible for reduced temperatures as low as 0.1 (or even below, see Ref. [9]), that corresponds some P-T conditions considered in this work. Thus, we are confident that structural properties and the immiscibility gap predicted in the present work are reliable.

One of the known shortcomings of semi-local GGA and meta-GGA level XC functionals is underestimation of

band gaps and related overestimation of dc conductivities. However, it was shown that semi-local XC functionals can provide reliable predictions (comparable to hybrid XC functional results) for the temperature of band gap closure (see Fig. S9 in the Supplemental Material for Ref. [8]. Given that in this work we are mostly interested in predictions of the metallization boundary of H-He mixtures, and not in absolute values of dc conductivity in the insulating regime, the choice of the semilocal KDT16 for Kubo-Greenwood calculations, instead of an expensive hybrid functional, is also justified.

ADDITIONAL RESULTS ON THE IMMISCIBILITY BOUNDARY

Figure 1a in the main text shows an example of typical snapshots of AIMD simulations at conditions inside the demixing domain, when we observe the demixing process directly in the simulation box. Helium-poor (gold) and helium-reach (cyan) regions can be identified by a visual observation. Nevertheless, a rigorous analysis of AIMD simulations and determination of the demixing/mixing transition temperature requires a quantitative measure of the demixing effects. From snapshots shown in Fig. 1a in the main text it is clear that the H-He interface in demixed two-phase state is a surface. Hence, the probability for He atom to find H atom at low distances is reduced as compared to the perfectly mixed system when the H-He interface is "volumetric".

The magnitude of the first peak in the H-He radial distribution function (RDF) along an isobar provides a sharp quantitative signature of H-He demixing and its transition to a perfectly mixed state. In sufficiently small H-He systems, demixing is avoided under all thermodynamic conditions [10]. Accordingly, the H-He "volumetric" (or bulk) interface enhances the probability of a He atom being close to an H atom. This process contributes to the first peak in the H-He RDF. At constant pressures, the magnitude of this peak is expected to decrease with an increase in temperature as a result of thermal expansion. In contrast, in large, demixed H-He systems, the first peak in the H-He RDF is expected to be significantly lower compared to a small, mixed system, as the bulk H-He interface reduces to a surface. The H-He demixing is most pronounced at the lowest temperatures but becomes less pronounced at higher temperatures until H-He is completely miscible. Accordingly, increasing the system's temperature increases the first peak in the H-He RDF. Subsequently, the first H-He RDF peak reaches the maximum upon perfect mixing and is expected to decrease with further temperature increase. Such a simple analysis of the H-He RDF behavior along isobars provides a single quantity that accurately and efficiently characterizes the demixing state independently of the shape of the He-rich regions.

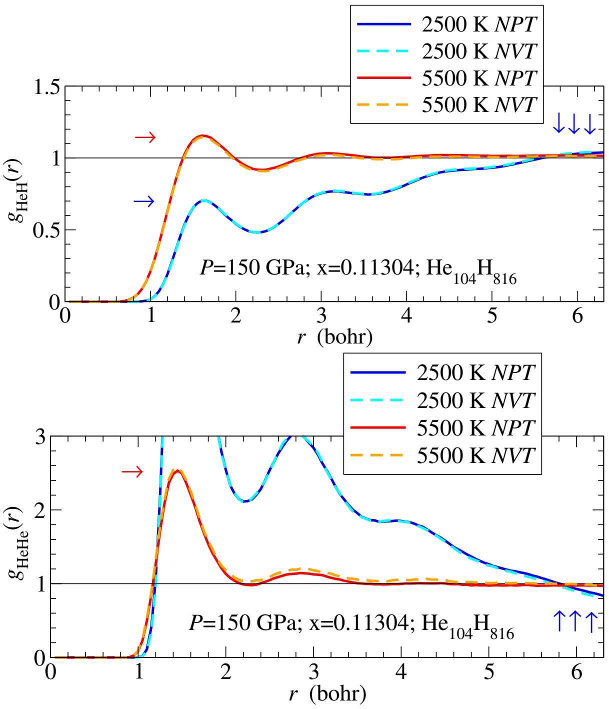

FIG. S1: Features of the He-H and He-He RDFs indicating the demixing process. Two examples are shown for a H-He ($He_{104}H_{816}$, x = 0.11304) mixture at a pressure of 150 GPa and temperatures of 2500 K and 5500 K corresponding to a demixed and mixed state, respectively. Solid curves correspond to *NPT* simulations, dashed curves result from complimentary *NVT* simulations.

Besides the first peak of the H–He radial distribution function (RDF) behaviour discussed in the main text, further qualitative features of H-H, He-He, and H-He RDFs indicating demixing are discussed in Refs. [11, 12]: (i) The probability for H/He atoms to be close to an atom of the same species at low distances is enhanced as compared to the perfectly mixed system; (ii) the probability for H/He atoms to be close to an atom of the same species at large distances is reduced, H-H and He-He RDFs at large distances drop below 1, while these values are equal to one in the case of near-perfect mixing; (iii) the probability of He atoms being close to H atoms at large distances is enhanced (greater than 1) compared to the perfect mixture. Figure S1 depicts the H-He and He-He RDFs for a pressures of 150 GPa and temperatures of 2500 and 5500 K, and illustrates some of these characteristics. In the present work, we observe the aforementioned changes in the RDF for thermodynamic conditions inside the demixing region. However, these features remain qualitative; it is difficult to quantify them as an accurate signature of the transition to a mixed state.

Our *NPT* results were validated by performing complimentary *NVT* simulations at selected *P*-*T* conditions. Since *NPT* predicts the average value of the material density ρ, we set up the corresponding *NVT* simulations such

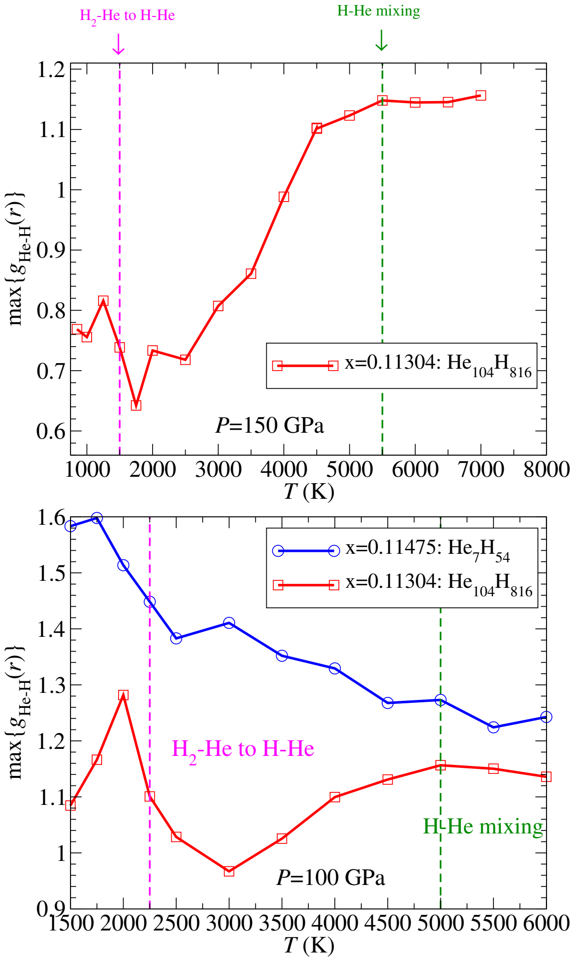

FIG. S2: Magnitude of the first H-He RDF peak as a function of temperature for pressures of 150 and 100 GPa. The vertical green dashed line indicates the transition to the perfectly mixed state of liquid H-He ($He_{104}H_{816}$, x = 0.11304) mixtures. Vertical magenta dashed lines depict the H_2-He to H-He transition.

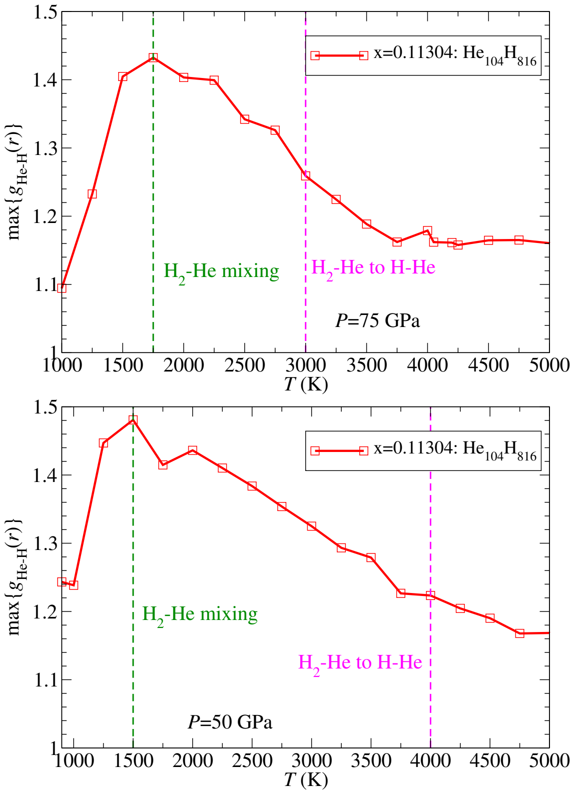

FIG. S3: Magnitude of the first H-He RDF peak as a function of temperature for pressures of 75 and 50 GPa. The vertical green dashed line indicates the transition to the perfectly mixed state of liquid H-He ($He_{104}H_{816}$, x = 0.11304) mixtures. Vertical magenta dashed lines depict the H_2-He to H-He transition.

that V corresponds to the NPT-predicted ρ value. For example, the predicted material density values at $P = 150$ GPa, and $T = 2500$ and 5500 K are $\rho_1 = 1.02564$ g/cm^3 and $\rho_2 = 0.94472$ g/cm^3 respectively. The average values of P from two complementary NVT for ρ_1 at 2500 K, and for ρ_2 at 5500K are 149.8 and 150.1 GPa respectively (i.e. identical to P in NPT). Importantly, the RDFs from NVT and NPT, compared in Fig. S1, are near-identical.

Figures S2-S3 show the magnitude of the first peaks of the H-He RDF as a function of temperature along the pressures of 150, 100, 75, and 50 GPa and He fraction x = 0.11304 (red curve). The H-He system transfers from a demixed to a mixed state once the curve reaches its maximum (green dashed line). Our findings for 100 GPa (Fig. S2 lower panel) are very similar to the results for 150 GPa (Fig. 1 in the main text and Fig. S2 upper panel) with two distinctions: (i) the magnitude of the first H-He RDF peak in the H_2-He mixture has a significantly larger increase from 1.08 to 1.28 for temperatures between 1500 and 2000 K; and (ii) a drop of the magnitude upon the dissociation of the H_2 subsystem (the complete dissociation corresponds to $\approx$ 2250 K, see corresponding discussions of Fig. S6 below) accompanied by almost simultaneous metallization. Inspecting the magnitude of the first H-He RDF peak for pressures of 75 and 50 GPa reveals a pronounced maximum at 1750 and 1500 K respectively, indicating the transition to a mixed state. The H_2 subsystem dissociation occurs already in the mixed state at 3000 and 4000 K for pressures of 75 and 50 GPa, respectively (see discussions of Fig. S7 below for details).

Moreover, we conducted simulations for higher He fractions x = 0.27522 and for pressures between 75 and 150 GPa. Figure S4 shows the magnitude of the first H-He RDF peak as a function of temperature along the pressures of 150 (upper panel) and 75 GPa (lower panel). The green dashed lines indicate the transition temperature from the demixed system to the mixed system (6500 and 2500 K for 75 and 150 GPa, respectively). At a pressure of 75 GPa the H_2 subsystem dissociates at $\approx$ 3750 K (see Fig. S10**c** below) once the system is mixed. These findings are similar to our simulations at a concentration

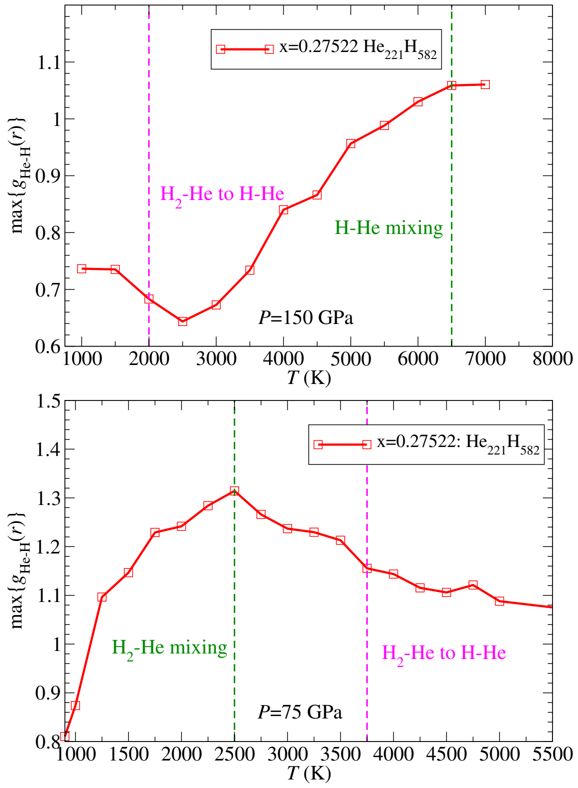

FIG. S4: Magnitude of the first H-He RDF peak as a function of temperature for pressures of 150 and 75 GPa. The vertical green dashed line indicates the transition to the perfectly mixed state of liquid H-He ($He_{221}H_{582}$, x = 0.27522) mixtures.

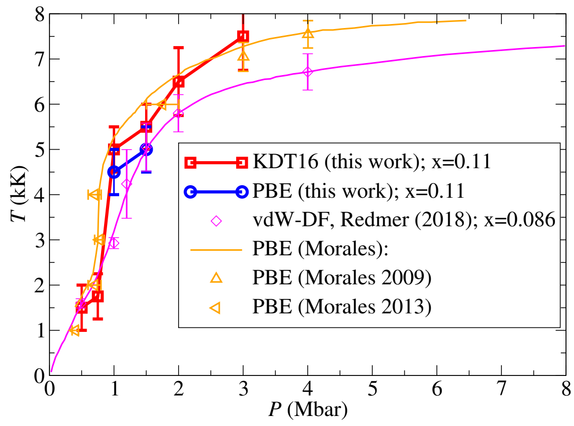

FIG. S5: He-H miscibility boundary as identified by the thermal KDT16 XC functional for $He_{104}H_{816}$ (x=0.11304) mixture. The ground-state PBE and vdW-DF XC predictions are shown for comparison.

of x = 0.11304 and a pressure of 50 GPa.

Figure S5 summarizes results for the H-He immiscibility boundary for $He_{104}H_{816}$ (x=0.11304) mixture. This Figure shows that simulations with the thermal KDT16 GGA XC (red squares) predict the boundary located about 500 K higher as compared to the present work simulations

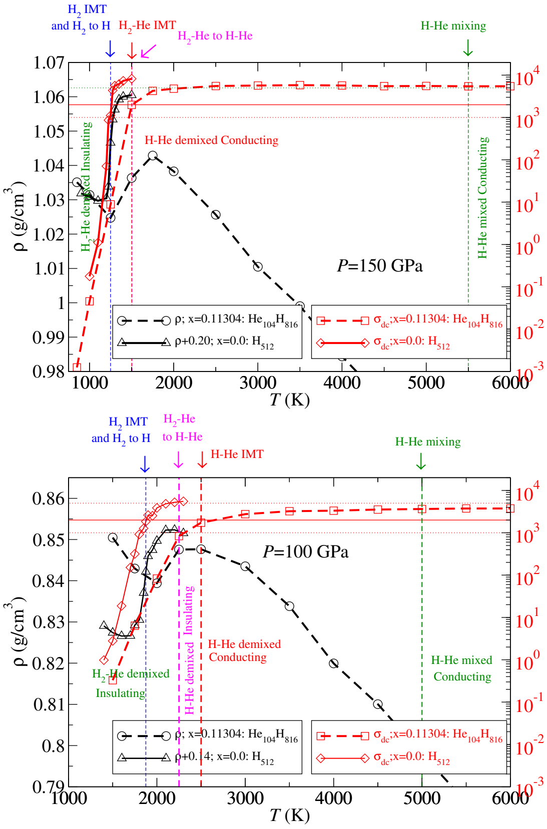

FIG. S6: Density and dc conductivity profiles for pressures of 150 and 100 GPa indicating temperatures of the H_2-He to H-He, IMT, and H-He demixing/mixing transitions of liquid He-H ($He_{104}H_{816}$, x = 0.11304) mixtures as predicted by DFT simulations with the thermal KDT16 GGA XC. The density of pure H (x=0.0) is shifted by a constant value for better visualization.

the ground-state PBE XC (blue circles). Our simulations also predict the immiscibility boundary systematically higher as compared to the simulations with ground-state vdW-DF XC indicating importance of the thermal XC effects at these thermodynamic conditions.

ADDITIONAL RESULTS ON THE STRUCTURAL AND CONDUCTING PROPERTIES

We calculated density profiles and dc conductivities of H-He mixtures along selected isobars to study the miscibility gap, structural properties, the dissociation transition from H_2-He to H-He, and the corresponding insulator-to-metal transition. Our findings for pressures of 150 (same as in the main text), 100, 75, and 50 GPa and for a He fraction of x = 0.11304 are presented in Figs. S6-S7

An increase in density and dc conductivity accompanies the dissociation of H_2 in the mixture and the pure hydrogen system. The density increase is consistent with

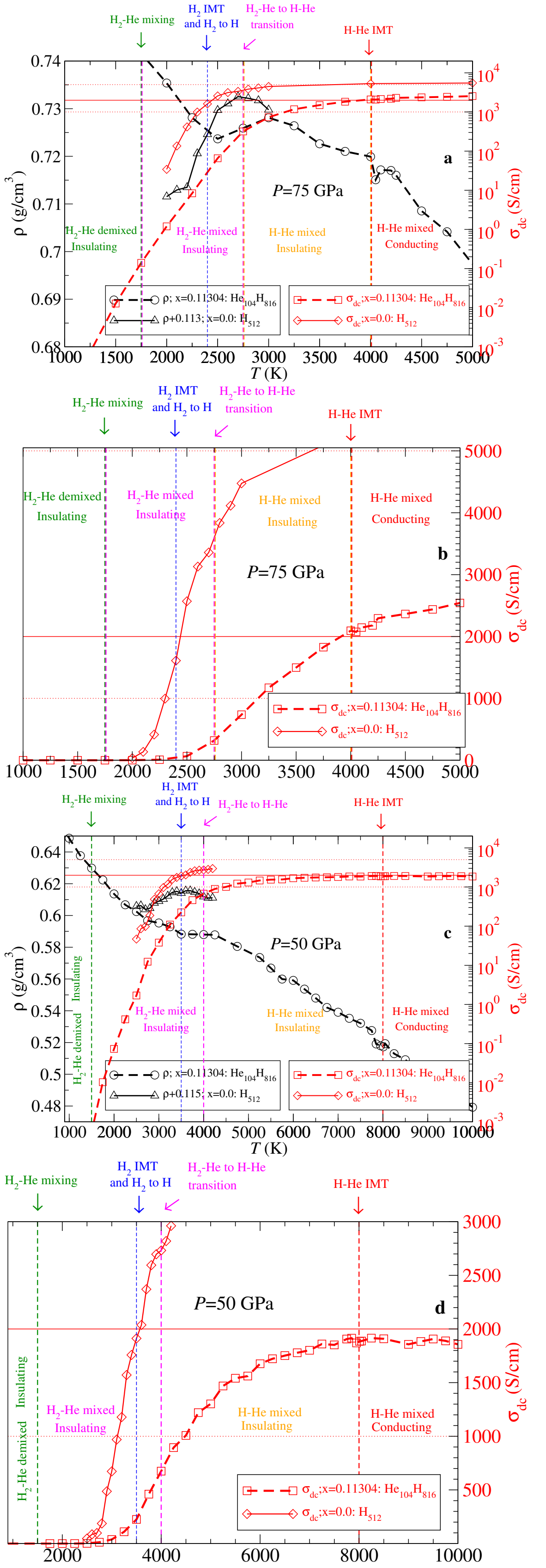

FIG. S7: Density and dc conductivity profiles for pressures of 75 and 50 GPa indicating temperatures of the H_2-He to H-He, IMT, and H-He demixing/mixing transitions of liquid He-H ($He_{104}H_{816}$, x = 0.11304) mixtures as predicted by DFT simulations with advanced thermal KDT16 XC. The density of pure H (x=0.0) is shifted by a constant value for better visualization.

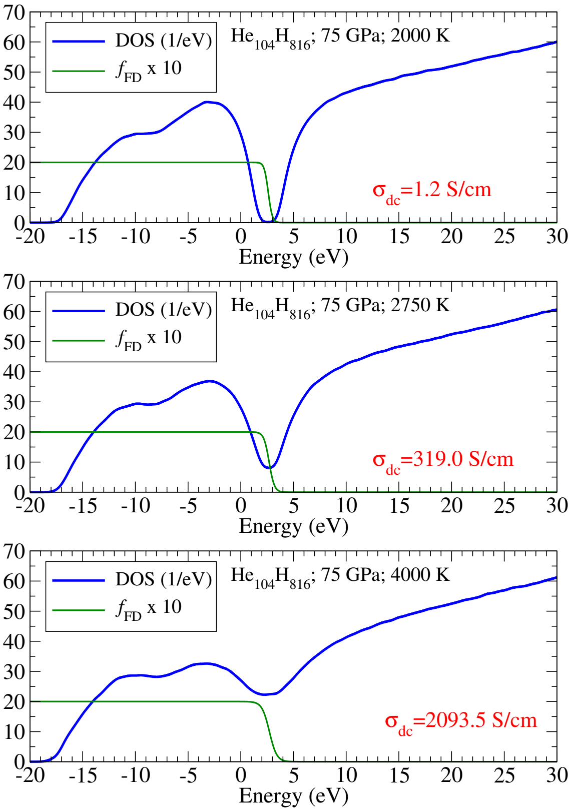

FIG. S8: Density of states (solid blue curve) and Fermi-Dirac occupations (solid green curve) for mixtures at $P = 75$ GPa and $T = 2000$ K (H_2-He mixed insulating), 2750 K (H-He mixed insulating upon H_2 subsystem dissociation), and at the IMT temperature 4000 K (H-He mixed conducting).

the drop in the magnitude of the first H-H RDF peak, indicating the dissociation process (see Fig. S11 below). However, a small fraction of He in the mixture strongly affects the dissociation and metallization processes as compared to the pure hydrogen system: (i) the density increase in the mixture becomes much smoother, eventually converting into a plateau at the lowest investigated pressure of 50 GPa; (ii) the dissociation process in mixtures is delayed by ≈ 250 K (or more at the lowest pressure) as a consequence of the H_2 bond strengthening [13]; (iii) the insulator-metal-transition (IMT) in the pure H system takes place essentially together with the dissociation, while the metallization temperature in mixtures, at pressures of ≥ 100 GPa, is higher as compared to the temperature of the H_2 subsystem dissociation.

The temperature offset of the metallization in H-He mixtures relative to the metallization of the molecular H_2 subsystem grows to several thousand Kelvins with a decrease in pressure. At a pressure of 100 GPa, the offset is ≈ 250 K, it increases to ≈ 1250 and ≈ 4000 K for pressures of 75 and 50 GPa, respectively (see Figs. S6 and S7).

Figure S8 shows the density of states (DOS) on the

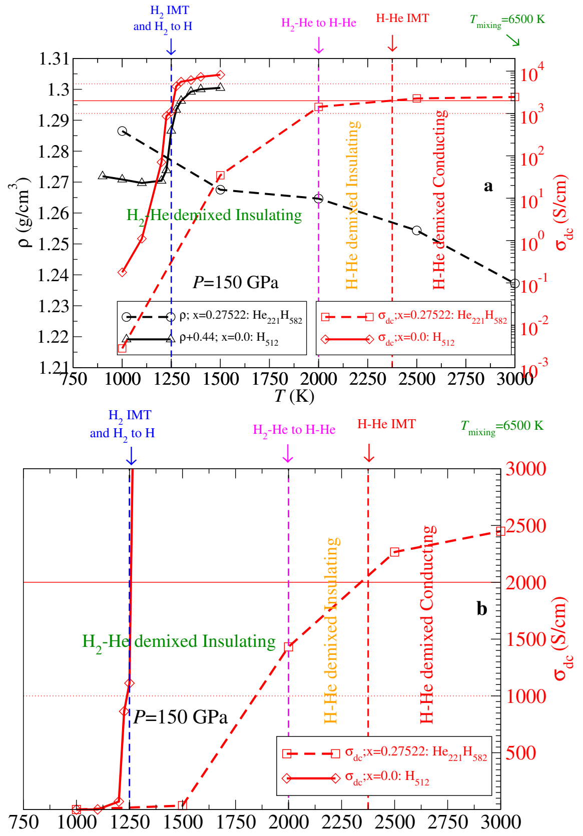

FIG. S9: Density and dc conductivity profiles for a pressure of 150 GPa indicating the temperatures of the H_2-He to H-He, IMT, and H-He demixing/mixing transitions of liquid He-H mixture ($He_{221}H_{582}$, x = 0.27522) as predicted by DFT simulations with advanced thermal KDT16 XC. The density of pure H (x=0.0) is shifted by a constant value for better visualization.

H-He mixture at $P = 75$ GPa and three representative temperatures, $T = 2000$ K when the system is a mixture of molecular H_2 and He, the DOS has a gap and the system is insulating. Upon dissociation of the H_2 subsystem at 2750 K the mixture exhibits a pseudogap (deep in DOS) and remains insulating. Eventually, upon IMT at $T = 4000$ K the gap closes, and the dc conductivity reaches the minimum metallic conductivity value of 2000 S/cm (see Fig. 5 in the main text for details of the system structure).

Figs. S9 and S10 show density profiles, conductivity profiles, temperatures of the immiscibility boundary, the H_2-He to H-He transition, the insulator-to-metal transition in the H-He mixtures for a He fraction of x = 0.27522 for pressures of 150, 100 and 75 GPa isobars. We find the offset of the metallization of H-He mixtures relative to the H_2-He to H-He transition for all investigated pressures. This offset is ≈ 375, 5000, and 6000 K for pressures of 150, 100, and 75 GPa, respectively. (REMARK: The insulator-to-metal transition temperature for pressures of 100 and 75 GPa is estimated by interpolation of the dc

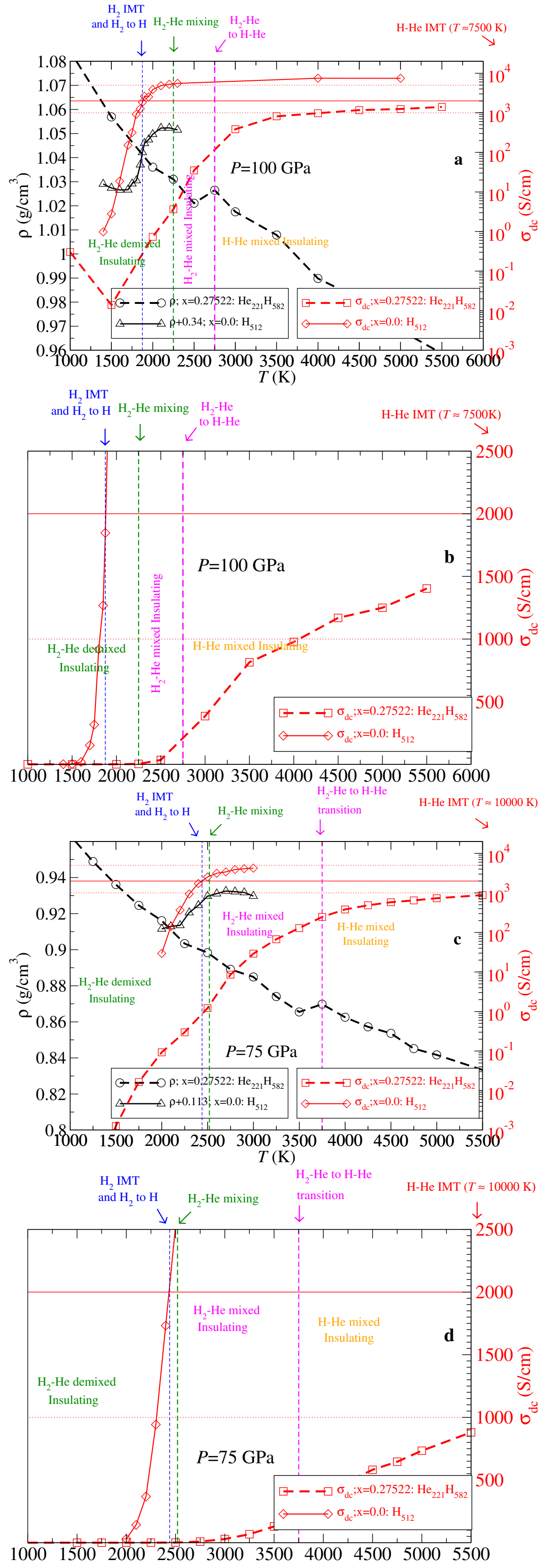

FIG. S10: Density and dc conductivity profiles for pressures of 100 and 75 GPa indicating temperatures of the H_2-He to H-He, IMT, and H-He demixing/mixing transitions of liquid He-H ($He_{221}H_{582}$, x = 0.27522) mixtures as predicted by DFT simulations with advanced thermal KDT16 XC. The density of pure H (x=0.0) is shifted by a constant value for better visualization.

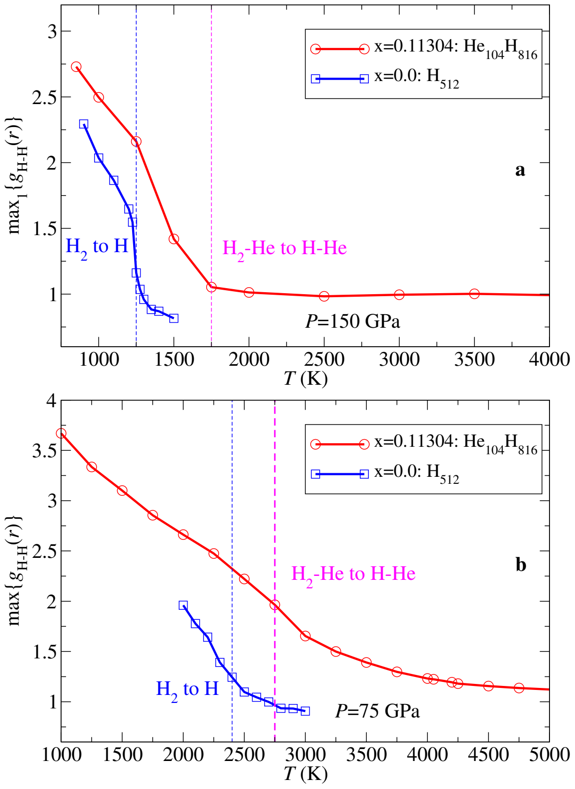

FIG. S11: Drop of the magnitude of the first H-H RDF peak for pressures of 150 and 75 GPa corresponding to the $H_2 \to H$ and H_2-He $\to$ H-He transitions in pure hydrogen and H-He mixtures for a helium fraction x = 0.11304. Vertical lines indicate the H_2 (sub-)system atomization.

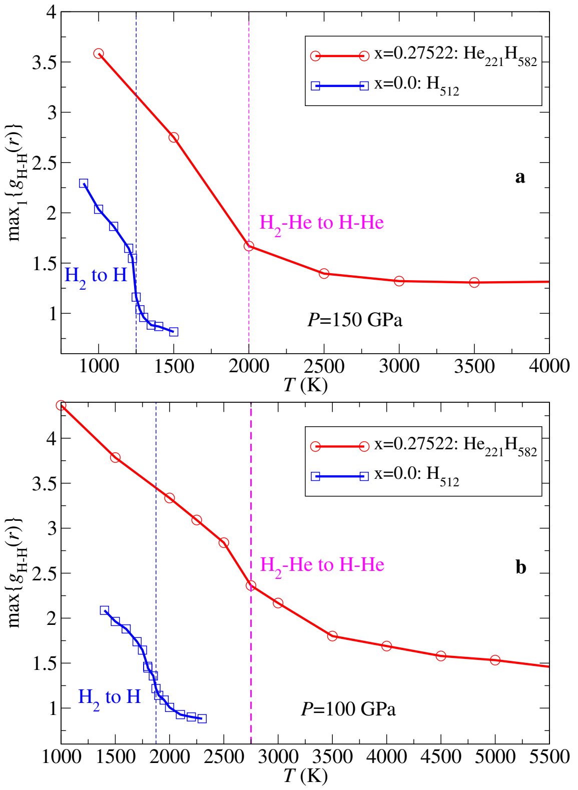

FIG. S12: Drop of the magnitude of the first H-H RDF peak for pressures of 150 and 75 GPa corresponding to the $H_2 \to H$ and H_2-He $\to$ H-He transitions in pure hydrogen and H-He mixtures for a helium fraction x = 0.27522. Vertical lines indicate the H_2 (sub-)system atomization.

conductivities shown in Fig. S10).

Figures S11 and S12 show the magnitude of the first H-H RDF peak as a function of temperature along selected pressures for He fractions x = 0.11304 and x = 0.27522. The magnitude drop in pure H and H-He mixtures corresponds to the H_2 (sub-)system dissociation. Note that these drops are sharp for pressures of 150 GPa but become smoother for lower pressures.

CONVERGENCE WITH RESPECT TO THE SYSTEM SIZE

Figure S13 presents the magnitude of the first H-He RDF peak for pressures of 150 GPa predicted by large-scale AIMD simulations for two different particle numbers employing the ground-state PBE XC. A comparison demonstrates that reducing the system size by a factor of two (from $He_{104}H_{816}$ to $He_{52}H_{408}$) does not alter the results for the H-He mixing temperature as predicted by the mechanical “signature” based on the H-He RDF features. Therefore, we conclude that our simulations are well-converged for predictions based on structural properties, i.e. for RDFs, immiscibility gap, and the H_2 subsystem atomization. Limited tests, not shown here, also demonstrated that systems with 1024 electrons are large enough for the convergence of transport properties.

REMARK: “large-scaling” of simulations usually is defined by the effective system size: the number of active electrons, or more accurately, the number of thermally occupied bands, and also by the simulation length. The two terms “large-scale” and “heavy/computationally expensive” are not interchangeable, a “large-scale” does not necessarily mean “heavy”, and vise-versa. One example is a long AIMD simulation on many thousands of atoms driven by an orbital-free DFT (OF-DFT) method which is probably not heavy (due to a favorable near-linear scaling of OF-DFT w.r.t. the system size) but is certainly a large-scale one. The increase in the effective system size by a factor of two increases the effective “large-scaling” by a factor of two as well, and not by a factor of eight according to the MKS computational cost scaling. The present work simulations are performed on the system size of 1024 electrons, the number of thermally occupied bands is up to 1100 in AIMD simulations, and up to 3300 bands employed in Kubo-Greenwood simulations. Simulations required unusually long AIMD trajectories, up to 40,000 MD steps. The simulations of

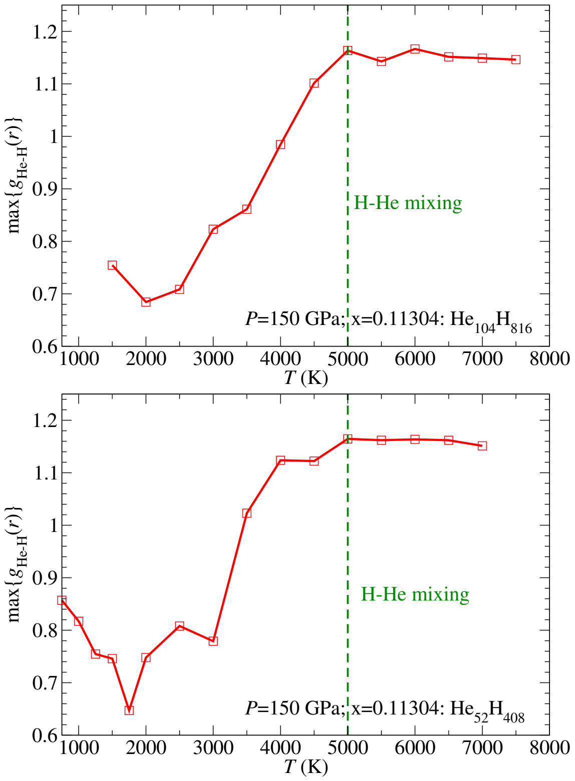

FIG. S13: Magnitude of the first H-He RDF peak for a pressure of 150 GPa. The vertical green dashed line indicates the transition to the perfectly mixed state of liquid H-He state. Simulations are conducted for systems with 1024 e^- ($He_{104}H_{816}$, x = 0.11304, upper panel) and 512 e^- ($He_{52}H_{408}$, x = 0.11475 (bottom panel) employing the ground-state PBE XC.

the present work are certainly qualified as "large-scale" ones.

* Electronic address: vkarasev@lle.rochester.edu

[1] G. Kresse and J. Furthmüller, Phys. Rev. B **54**, 11169 (1996), URL https://urldefense.proofpoint.com/v2/url?u=https-3A__link.aps.org_doi_10.1103_PhysRevB.54.11169&d=DwIGaQ&c=sJ6xIWYx-zLMB3EPkvcnVg&r=v9go-pOEKfD81b69rRPRGw&m=80sjS5LBcHOhWvs1SDSCHeAP_GXweo-Ow2P-whiVqwU&s=_JmcxDe_deLJi0-PV4FWneAz86zMv1p9BT5dm42VMCc&e=.

[2] G. Kresse and D. Joubert, Phys. Rev. B **59**, 1758 (1999), URL https://urldefense.proofpoint.com/v2/url?u=http-3A__link.aps.org_doi_10.1103_PhysRevB.59.1758&d=DwIGaQ&c=sJ6xIWYx-zLMB3EPkvcnVg&r=v9go-pOEKfD81b69rRPRGw&m=80sjS5LBcHOhWvs1SDSCHeAP_GXweo-Ow2P-whiVqwU&s=peXOvuE5pkdwur7q4pZTeKwCLyOYCBGuafpPoorCUyI&e=.

[3] V. V. Karasiev, J. W. Dufty, and S. B. Trickey, Phys. Rev. Lett. **120**, 076401 (2018), URL https://urldefense.proofpoint.com/v2/url?u=https-3A__link.aps.org_doi_10.1103_PhysRevLett.120.076401&d=DwIGaQ&c=sJ6xIWYx-zLMB3EPkvcnVg&r=v9go-pOEKfD81b69rRPRGw&m=80sjS5LBcHOhWvs1SDSCHeAP_GXweo-Ow2P-whiVqwU&s=9P5hABD9gNtOBXfc0iCqPpbXPFRjbiSfYjIT6rTUKOY&e=.

[4] A. Baldereschi, Phys. Rev. B **7**, 5212 (1973), URL https://link.aps.org/doi/10.1103/PhysRevB.7.5212.

[5] V. V. Karasiev, J. Hinz, S. X. Hu, and et al., Nature **600**, E12 (2021).

[6] J. P. Perdew, K. Burke, and M. Ernzerhof, Physical review letters **77**, 3865 (1996).

[7] L. Calderín, V. Karasiev, and S. Trickey, Computer Physics Communications **221**, 118 (2017), ISSN 0010-4655, URL https://www.sciencedirect.com/science/article/pii/S0010465517302539.

[8] J. Hinz, V. V. Karasiev, S. X. Hu, M. Zaghoo, D. Mejía-Rodríguez, S. B. Trickey, and L. Calderín, Phys. Rev. Res. **2**, 032065 (2020), URL https://link.aps.org/doi/10.1103/PhysRevResearch.2.032065.

[9] V. V. Karasiev, S. X. Hu, M. Zaghoo, and T. R. Boehly, Phys. Rev. B **99**, 214110 (2019), URL https://link.aps.org/doi/10.1103/PhysRevB.99.214110.

[10] M. Schöttler and R. Redmer, Phys. Rev. Lett. **120**, 115703 (2018), URL https://link.aps.org/doi/10.1103/PhysRevLett.120.115703.

[11] W. Lorenzen, B. Holst, and R. Redmer, Phys. Rev. B **84**, 235109 (2011), URL https://link.aps.org/doi/10.1103/PhysRevB.84.235109.

[12] B. Militzer, Phys. Rev. B **87**, 014202 (2013), URL https://link.aps.org/doi/10.1103/PhysRevB.87.014202.

[13] J. Vorberger, I. Tamblyn, B. Militzer, and S. A. Bonev, Phys. Rev. B **75**, 024206 (2007), URL https://link.aps.org/doi/10.1103/PhysRevB.75.024206.
